# Telesurgery in Brazil: Opportunities, Responsibilities, and the Path Ahead

**DOI:** 10.1590/S1677-5538.IBJU.2025.0517

**Published:** 2025-09-15

**Authors:** Marcio Covas Moschovas, Alexandre Pompeo, Gustavo Cardoso Guimarães, Renato Almeida Rosa de Oliveira, Vipul Patel

**Affiliations:** 1 AdventHealth Global Robotics Institute Kissimmee FL United States AdventHealth Global Robotics Institute, Kissimmee, FL, United States; 2 University of Central Florida Orlando FL United States University of Central Florida - UCF, Orlando, FL, United States; 3 Hospital do Coracão São Paulo SP Brasil Hospital do Coracão – HCOR, São Paulo, SP, Brasil; 4 Beneficência Portuguesa de São Paulo São Paulo SP Brasil Beneficência Portuguesa de São Paulo – BP, São Paulo, SP, Brasil

## INTRODUCTION

Telesurgery is no longer a distant concept but rather a rapidly evolving reality in surgical innovation. The landmark Lindbergh operation in 2001 served as an early proof of principle, demonstrating that surgery could be performed across continents through a remote connection (1). However, only in recent years has telesurgery become a clinical reality, made possible by the advent of high-speed 5G networks, reliable fiber-optic connections, and the development of new robotic platforms that can integrate these technologies (1-9). Worldwide, several procedures have been successfully performed under research protocols, validating both safety and feasibility (5-8, 10-15). In Brazil, however, the incorporation of telesurgery into clinical practice remains in its early stages, still surrounded by ethical, legal, and technical considerations that must be carefully addressed (15). The motivation for this letter arises from the emergence of new robotic platforms with telesurgery capabilities, such as the MicroPort Toumai in the Brazilian market. At the same time, standard platforms like Intuitive and Medtronic also showed their telesurgery capabilities during the Society of Robotic Surgery (SRS) 2025 in Strasbourg. These advances have profound humanitarian implications: they not only promise to reduce surgical complications and improve patient access but also hold the potential to transform how we teach and disseminate robotic surgery across vast and underserved regions.

From a legal perspective, the Brazilian Federal Medical Council (CFM) has taken important first steps in establishing a framework for telesurgery in 2022. CFM Resolution No. 2,311/2022 requires that the remote surgeon hold appropriate specialist certification and prior robotic training, while also mandating the presence of an equally qualified local surgeon in the operating room. This action is crucial to ensure patient safety and facilitate immediate intervention in case of communication failure or robotic malfunction. In parallel, data security and compliance with the General Data Protection Law (LGPD) are indispensable, given the sensitive transmission of patient information across networks. ANVISA's (Agência Nacional de Vigilância Sanitária) role in certifying robotic systems and supervising medical devices also anchors the legal basis for telesurgery in Brazil. These multiple layers of regulation underscore that telesurgery cannot be approached as a purely technological experiment; it is an act of medicine with strict professional, institutional, and legal responsibilities.

Ethical considerations are equally significant in the practice of remote surgery (9, 16-20). Informed consent must be specific and transparent, ensuring that patients fully understand the nature of remote surgery, the technology involved, potential risks, and alternatives in case of technological impairments during the procedure (18). Responsibility in telesurgery is inherently distributed: the remote surgeon guides the procedure, the local surgeon guarantees safety at the bedside, and institutions are accountable for infrastructure, connectivity, and team readiness. Without clear ethical guidelines defining the scope of each actor's duty, patient safety and professional accountability risk being undermined. In this scenario, due to the lack of international guidelines, we believe that the best practice of telesurgery in Brazil and worldwide at this moment should follow a strict study protocol under the oversight of multi-institutional IRBs (Internal Review Boards), allowing the collection of standardized data, metrics, and the evaluation of clinical, ethical, and economic benefits before wider implementation (3, 4, 18, 21, 22).

Beyond the legal and ethical challenges, telesurgery carries a profound humanitarian potential, and Brazil, as the fifth-largest country in the world, faces vast geographic disparities in healthcare access (21, 23, 24). Patients in remote or underserved regions often lack access to high-complexity surgical care, and telesurgery offers a pathway to mitigate these inequalities. By enabling highly specialized procedures to be performed remotely, telesurgery could democratize access to cutting-edge care, provided that infrastructure and resources are distributed equitably. For the "Sistema Único de Saúde" (SUS), this innovation has the potential to bring expertise to areas where it has historically been absent (21). From the very beginning, however, we must ensure that this technology is established in a way that serves the entire population, rather than being restricted to a few privileged centers. In this scenario, the entry of new robotic platforms into the market may play a decisive role by reducing the costs of technology and enabling access for more patients, particularly those treated within the SUS.

In this context, a common criticism of telesurgery is that it requires the presence of a qualified surgeon at the patient's side, which at first look may appear to limit its economic justification. However, this interpretation overlooks the central concept of telesurgery as a long-term strategy for building local expertise. In the initial phases, experienced surgeons may indeed need to travel to remote sites; however, over time, these local teams acquire the necessary skills to complete procedures, including open conversions if required, independently (6, 10). The value of the remote expert lies not in replacing the local team but in being available to supervise multiple centers on the same day, guiding critical portions of complex cases, and accelerating the learning curve of surgeons in training. This model creates a multiplier effect, allowing one expert to support multiple surgeons and patients across large geographic regions, thereby alleviating the burden of repeated travel.

When evaluated from a broader healthcare perspective, telesurgery should extend beyond robotics to areas such as cardiac and neurosurgery. Considering that thousands of patients in Brazil suffer strokes and myocardial infarctions every year, the need for timely specialized care in remote areas is urgent, especially in the public healthcare (SUS). Patients in these regions who experience strokes, heart attacks, or other acute conditions often face death or lifelong sequelae due to the lack of immediate expert intervention. The long-term costs of chronic disability, including physical rehabilitation, loss of productivity, hospital readmissions, and premature mortality, far exceed the investment required for a robust telesurgery program. By enabling expert input where it is most urgently needed, telesurgery can reduce complications, improve outcomes, and deliver cost savings for the entire health system. The same concept can be applied when considering biopsies and early disease diagnosis that require a tissue sample from patients in remote areas.

Another important humanitarian application of telesurgery lies in its role as a tool for teleproctoring and surgical teaching (5, 7, 15, 25-28). Brazil has several talented surgeons and trainees who are eager to expand their expertise in robotic surgery, but geographic distance often limits access to high-quality mentorship. In this scenario, remote proctoring offers a solution, allowing experienced surgeons to guide and supervise procedures in real time without the constant need for travel. This not only accelerates the learning process but also reduces the significant logistical burden of repeatedly transporting patients and families to referral centers or flying experts across the country. In a continental-size country such as Brazil, where a single domestic flight may take over six hours in addition to long layovers and transfers, the barriers imposed by distance are a major obstacle to surgical innovation and equitable care. Telesurgery and teleproctoring, by overcoming these barriers, provide a sustainable model for training, knowledge dissemination, and ultimately, the democratization of advanced surgical techniques.

The future of telesurgery in Brazil will depend on moving away from the notion that it can be implemented by a single surgeon in partnership with a technology company, or that its purpose is to increase competition and replace physicians. Such concepts should be firmly discouraged. Telesurgery, by definition, is a collective effort that requires the coordinated participation of all responsible stakeholders: the Federal (CFM) and Regional Medical Councils (CRM), surgical societies, hospital administrators, government agencies, telecom providers, technology and robotic companies, cybersecurity experts, and surgeons. Only with this collaborative mindset can telesurgery be developed in a safe, ethical, and truly transformative way for Brazilian healthcare.

Telesurgery in Brazil is no longer just a technological promise; it is a responsibility. It's safe adoption requires clear ethical and legal frameworks, strict study protocols (IRBs), and above all, teamwork (4). This is not the project of one surgeon or one company, but a collective effort involving medical councils, surgical societies, hospitals, government agencies, technology providers, and surgeons. If built with these standards, telesurgery can expand access to care, reduce costs, and create new opportunities for teaching and training. In a country as large and diverse as Brazil, it represents not only innovation but also a humanitarian obligation to bring high-quality surgery to all patients, regardless of their location. The future of telesurgery depends on a collective effort, supported by evidence and social commitment, so that this innovation may become a valid instrument of equity and transformation within the Brazilian healthcare system.

## Data Availability

Not applicable
